# Treadmill training and physiotherapy similarly improve dual task gait performance: a randomized-controlled trial in Parkinson’s disease

**DOI:** 10.1007/s00702-022-02514-4

**Published:** 2022-06-13

**Authors:** Heiko Gaßner, Elmar Trutt, Sarah Seifferth, Jana Friedrich, Diana Zucker, Ziad Salhani, Werner Adler, Jürgen Winkler, Wolfgang H. Jost

**Affiliations:** 1 Department of Molecular Neurology, Universitätsklinikum Erlangen, Molekulare Neurologie, Friedrich-Alexander-Universität Erlangen-Nürnberg (FAU), Schwabachanlage 6, 91054 Erlangen, Germany; 2 Parkinson-Klinik Ortenau, Wolfach, Germany; 3 Institut für Medizininformatik, Biometrie und Epidemiologie, FAU Erlangen-Nürnberg, Erlangen, Germany; 4 Fraunhofer IIS, Fraunhofer Institute for Integrated Circuits IIS, Am Wolfsmantel 33, 91058 Erlangen, Germany

**Keywords:** Parkinson’s disease, Gait analysis, Treadmill training, Physiotherapy, Dual task, Wearable sensors

## Abstract

Motor-cognitive dual tasks are used to investigate the interplay between gait and cognition. Dual task walking in patients with Parkinson’s disease (PD) results in decreased gait speed and more importantly in an increased fall risk. There is evidence that physical training may improve gait during dual task challenge. Physiotherapy and treadmill walking are known to improve single task gait. The aim of this study was to investigate the impact of individualized physiotherapy or treadmill training on gait during dual task performance. 105 PD patients were randomly assigned to an intervention group (physiotherapy or treadmill). Both groups received 10 individual interventional sessions of 25 min each and additional group therapy sessions for 14 days. Primary outcome measure was the dual task gait speed. Secondary outcomes were additional gait parameters during dual task walking, UPDRS-III, BBS and walking capacity. All gait parameters were recorded using sensor-based gait analysis. Gait speed improved significantly by 4.2% (treadmill) and 8.3% (physiotherapy). Almost all secondary gait parameters, UPDRS-III, BBS, and walking capacity improved significantly and similarly in both groups. However, interaction effects were not observed. Both interventions significantly improved gait in patients with mild to moderate PD. However, treadmill walking did not show significant benefits compared to individualized physiotherapy. Our data suggest that both interventions improve dual task walking and therefore support safe and independent walking. This result may lead to more tailored therapeutic preferences.

## Introduction

Dual task walking defined as walking while carrying out another task of interfering with walking, e.g., talking or carrying a tray, is essential for daily living. It has a huge impact on independence and therefore quality of life (QoL). In healthy adults as well as in patients with Parkinson’s disease (PD), gait and postural stability are negatively impacted while performing a dual task (Gassner et al. 2017; O'Shea et al. 2002; Woollacott and Shumway-Cook 2002). As PD patients show major motor deficits, such as disturbed postural stability, increased gait variability, and cognitive decline in more advanced stages, they are especially vulnerable to dual tasks (Brauer et al. 2011; Woollacott and Shumway-Cook 2002; Yogev et al. 2005; Yogev-Seligmann et al. 2012). The impact on gait performance increases with age (Woollacott and Shumway-Cook 2002) and disease progression in PD (Rochester et al. 2014).

In particular, dual tasking in PD patients reduced gait velocity and step length (Bond and Morris 2000; Galletly and Brauer 2005; Gassner et al. 2017; Morris et al. 1996; O'Shea et al. 2002; Yogev et al. 2005), but increased gait asymmetry (Yogev et al. 2007). Especially gait parameters linked to postural control were affected (Rochester et al. 2014). Increased fall risk is linked to dual task walking as well (Brauer et al. 2011; Plotnik et al. 2011; Yogev et al. 2005; Yogev-Seligmann et al. 2012), as is an increase in freezing episodes under challenging conditions (Spildooren et al. 2010).

Sensor-based gait analysis has attracted major attention over the last years. Most of the studies previously performed already used sensor-based technologies to record gait parameters. Sensors are able to capture metric parameters regularly inaccessible or very time-consuming for clinicians (Espay et al. 2016), e.g., gait variability which is increased by dual tasking (Lord et al. 2011). Especially, wearable sensors provide a good mean to record clinically relevant gait parameters in different settings. They have been proven to be technically valid and reliable in achieving objective and clinically relevant characteristics of gait (Kluge et al. 2017; Schlachetzki et al. 2017). In the present study, sensor-based gait parameters serve as digital, quantitative, and objective outcome measure.

There is evidence that gait during dual task conditions may be improved by training (Brauer et al. 2011; Geroin et al. 2018; Strouwen et al. 2019; Yogev-Seligmann et al. 2012). Studies that examined effects on dual tasks mostly used special dual task training consisting of a walking component and a cognitive component as intervention (“practice makes perfect”) (Brauer et al. 2011; Strouwen et al. 2017; Yogev-Seligmann et al. 2012). Interventions lasted for 4–6 weeks (Brauer et al. 2011; Geroin et al. 2018; Strouwen et al. 2017; Yogev-Seligmann et al. 2012) and resulted in higher gait speed and stride length as well as cadence (Brauer and Morris, 2010; Geroin et al. 2018; Strouwen et al. 2019, 2017; Yogev-Seligmann et al. 2012). Especially motor learning principles and task-specific training led to improvements (Yogev-Seligmann et al. 2012). In the present study we investigated the ‘Parkinson’s Disease Multidisciplinary Rehabilitation’ program established in Germany defined as intervention that lasts 14 days. There are several studies in various exercise fields (e.g., cue training, amplitude oriented exercises or treadmill walking) suggesting that short intervention periods (around 2 weeks but for some even a single session) positively impact motor symptoms in Parkinson’s disease (Bello et al. 2008; Ebersbach et al. 2015; Klamroth et al. 2016; Lehman et al. 2005; Nieuwboer et al. 2007; Thaut et al. 1996).

Structured physiotherapy is commonly known to be effective in improving motor symptoms and gait in patients with PD (Armstrong and Okun, 2020; Paz et al. 2019; Pellecchia et al. 2004; Radder et al. 2020). Treadmill training as an instrumented training approach significantly improved gait (Gassner et al. 2019; Paz et al. 2019; Radder et al. 2020) as well as the motor part of the Unified Parkinson’s Disease Rating Scale (UPDRS-III) (Gassner et al. 2019; Paz et al. 2019). In direct comparison, treadmill training was more effective in improving single task gait performance than standardized physiotherapy without treadmill walking (Mehrholz et al. 2015; Paz et al. 2019).

However, to the best of our knowledge, there is a lack of studies assessing the influence of structured physiotherapy and treadmill training on dual task performance as an important factor of safe and independent walking in everyday life.

Therefore, the aim of this study was to investigate the impact of individualized physiotherapy and treadmill training on gait during dual task performance. Gait parameters as objective outcomes were recorded using a sensor-based gait system.

## Subjects and methods

### Study cohort

**Fig. 1:**
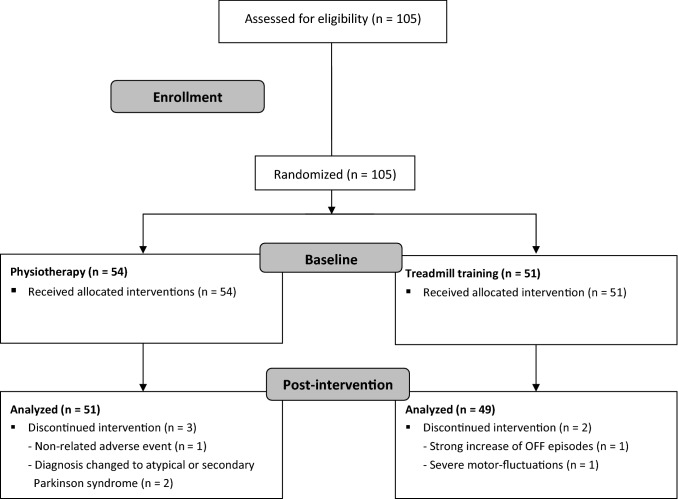
Flow diagram

We enrolled 105 patients diagnosed with PD (Fig. 1) as defined by the MDS-Criteria by Postuma et al. (Postuma et al. 2015). Inclusion criteria further consisted of age between 30 and 90 years and Hoehn and Yahr disease stage between I and III. Furthermore, patients had to be able to walk on a treadmill for 25 min without using handrail and needed to be cognitively able to perform the required tasks. Exclusion criteria included atypical or secondary Parkinson syndromes, severe freezing episodes, profound motor fluctuations or end-of-dose phenomenon, acute orthopedic gait impairment, central and peripheral paresis, extreme axial deformities as camptocormia, history of dementia, acute cardiac impairment, history of falling or acute psychiatric symptoms. All patients were enrolled at the Movement Disorders Center Ortenau, Wolfach, Germany. The flow diagram illustrating the recruiting process is presented in Fig. 1. Demographics and clinical characteristics of the study population at baseline are summarized in Table 1. Both groups were similar in terms of age, gender, height, and weight and show comparable clinical characteristics at baseline, such as LEDD (Levodopa equivalent daily dose), H&Y stage (Hoehn and Yahr), UPDRS-III (Unified Parkinson Disease Rating Scale, part III), and MoCA (Montreal Cognitive Assessment). This study was approved by the local ethics committee (reference number: F-2019-052, Landesärztekammer Baden-Württemberg, Stuttgart, Germany), and participants gave written informed consent according to the Declaration of Helsinki. This clinical study was registered in the German Clinical Trial Register under trial registration number DRKS00018841.

**Table 1:** Patient characteristics at baseline

	Treadmill group (*n* = 49)	Physiotherapy group (*n* = 51)	*p* value
	mean ± SD	mean ± SD	
Age, years	60.5 ± 9.1	61.7 ± 8.1	0.481
Gender, male/female	37/12	37/14	0.736^a^
Height, m	1.76 ± 0.08	1.74 ± 0.09	0.252
Weight, kg	84.1 ± 14.8	81.2 ± 13.1	0.311
LEDD, mg/d	566.7 ± 342.1	539.1 ± 323.6	0.679
H&Y stage	2.5 ± 0.5	2.5 ± 0.6	0.773
UPDRS-III Baseline	26.1 ± 7.77.7	23.9 ± 6.9	0.123
MoCA	26.6 ± 3.7	27.5 ± 2.8	0.156

UPDRS-III: Motor score of the Unified Parkinson’s disease rating scale MoCA: Montreal Cognitive Assessment

^a^Chi-Square Test; *LEDD* Levodopa equivalent daily dose, H&Y: Hoehn and Yahr disease stage

Patients (*n* = 105) were randomly allocated to one of the interventional approaches. We used a computer-generated block-randomization (block size changed randomly between 4 and 6) stratified by gender and H&Y stage (H&Y 1–2 and H&Y 2.5–3.5). Participants were informed that they receive one of two different treatment interventions; however, they were not aware of the difference between study arms or the research question. Both groups received 10 individualized exercise sessions of 25 min each as well as 11 sessions of group therapy with varying durations as described below, over the course of 14 days which is the defined time frame of the ‘Parkinson’s Disease Multidisciplinary Rehabilitation’ program established in Germany. In total, both therapy intervention groups received a comparable number of 250 min (individualized exercise) + 320 min (group exercise) = 570 min of therapy. All exercises were conducted under supervision of equally experienced physiotherapists, during individual exercises patients received one by one supervision.

Group 1 (“Treadmill”) received 8 treadmill walking sessions that were constructed of 5 × 5 min intervals referring to studies that investigated an immediate effect of treadmill walking on gait (Bello et al. 2008; Klamroth et al. 2016). The protocol followed was: (1) 5 min familiarization, (2) 5 min preferred walking speed, (3) 5 min slow walking speed, (4) 5 min preferred walking speed, (5) 5 min slow walking speed. On a walking speed scale of 1 (walking is not possible without handhold) to 10 (no problems), patients were instructed to choose a self-selected walking speed of 4–6 (indicating medium challenge for preferred and slow speed) on the treadmill.

Preferred and slow walking speeds varied over the intervention period due to training progression or personal well-being. Slow speed (defined as self-selected substantial reduction of preferred speed) was included in the training protocol to not overburden the participants and to train gait variability instead of walking on the same speed during the entire session. Furthermore, Group 1 received two sessions of physiotherapy during the intervention period (25 min each). Group 2 (“Physio”) received 8 physiotherapy and two endurance training sessions (all interventions of Group 2 did not include treadmill walking). The individualized physiotherapy intervention focused on postural perceptions, strategies to improve change of body positions (transfers), reactive, sensory, and anticipatory balance training as well as exercises to improve coordination and flexibility.

Both groups received group therapy sessions defined as components of the ‘Parkinson’s Disease Multidisciplinary Rehabilitation’ program: 2 × Tai Chi (50 min), 2 × Nordic Walking (90 min), 4 × medical exercise therapy using strength training equipment (100 min), 2 × stretching (30 min), and 1 × strength training for abdomen and back (50 min) (Ferrazzoli et al. 2018; Monticone et al. 2015; Rochester and Espay 2015). Furthermore, four sessions of occupational and four of speech therapy were part of the treatment regime.

### Outcomes

Assessments were conducted at baseline and after the intervention period of 14 days. Primary outcomes included gait speed and clinically relevant gait parameters, such as stride length and swing time, under dual task condition. Secondary outcomes were the UPDRS-III (Fahn and Elton 1987) and the Berg Balance Scale (BBS) (Scherfer et al. 2006) both rated by trained movement disorder specialists. Furthermore, the Montreal Cognitive Assessment (MoCA) (Nasreddine et al. 2005) was performed to screen for cognitive impairment.

### Walking tasks and instrumented gait analysis

Participants performed 2 × 10 m walking tests in single (ST) as well as dual task (DT) condition. ST included walking in self-selected comfortable walking speed over 20 m including a 180° turn after 10 m. In DT condition, subjects were asked to simultaneously walk and count backwards in steps of three, starting at 100. PD patients were not instructed to prioritize the walking or counting task, assuming that both tasks were performed with equal attention. DT costs were calculated as a measure of the effect of the cognitive task on the walking task (Gassner et al. 2017). The *2-Minute Walk Test* was performed to measure the patients’ walking capacity (Stewart et al. 1990). Participants walked on a 25-m long floor and turned around a cone at the end of each walking bout. They were instructed to walk as far as possible within 2 min without any additional motivation provided during the test.

Sensor-based gait patterns were recorded using the gait analysis systen *Mobile GaitLab* (Portabiles HealthCare Technologies, Erlangen, Germany) (Ullrich et al. 2021). Spatio-temporal gait parameters were extracted from sensor signals as described (Barth et al. 2015; Gassner et al. 2017; Rampp et al. 2013). Parameters representing gait variability were not included in the data analysis due to the fact that a larger number of strides would be necessary for a robust analysis of the coefficient of variance (Galna et al. 2013). Patients wore two IMU sensors attached in instep position on standardized types of shoes. Sensors shared a common time axis (left–right synchronized) (Roth et al. 2018) and each incorporated a 3D- accelerometer (range ± 16 g) as well as a 3D-gyroscope (range ± 2000 deg/s). Data were recorded at a sampling rate of 102.4 Hz.

Dual tasking results in a reduced performance in one or both concurrently performed tasks. Dual task costs were usually be calculated to detect the decline in gait parameters in comparison to single task walking. We calculated dual task costs using the following formula (Gassner et al. 2017; Rochester et al. 2014)

$$DT\,{\text{costs}}\,[\% ] = \frac{{(DT\,{\text{gait}}\,{\text{parameter}} - ST\,{\text{gait}}\,{\text{parameter}})}}{{ST\,{\text{gait}}\,{\text{parameter}}}} \times 100$$

### Statistical analysis

Gait parameters were evaluated by performing an ANOVA for repeated measures. We determined time (i.e., difference from baseline to post measure) and group as our main effects and investigated the interaction of time and group. Normality of data was tested by Kolmogorov–Smirnov test.

Sphericity was assumed, homogeneity of error variances was tested by Levene test, and homogeneity of covariance matrices was confirmed by Box test. Due to balanced group sizes, homogeneity was assumed for all gait parameters under the different conditions. For within-factor time (2,1) and between-factors group, we used a correction of the confidence interval by Bonferroni.

The clinical outcomes were evaluated using an ANOVA for repeated measures as well, with ‘time’ as within- and ‘group’ as between-factor. For all parameters, partial eta-squared was used as the dominant effect size. Explorative data analysis was performed for gender and added as a covariate to the repeated-measures ANOVA later. All statistical analyses were performed using SPSS software package version 24 (IBM Corp. Released 2016. IBM® SPSS® Statistics for Windows, Version 24.0.0.2, Armonk, NY, USA: IBM Corp.).

## Results

Main effects of time and group as well as interaction of time and group were evaluated for each of the assessed outcomes.

### Primary outcome—gait velocity

**Fig. 2:**
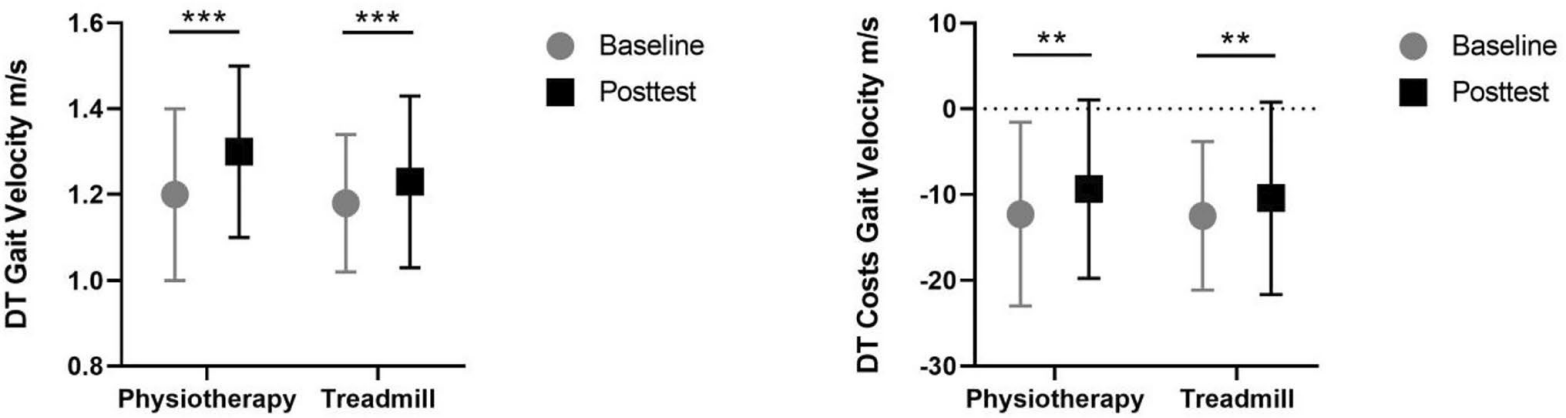
Primary outcome measure: change of dual task (DT) gait velocity (left) and dual task costs for gait velocity (right)

Most importantly, both interventional approaches resulted in a significant improvement over time in DT gait velocity (*p* < 0.001) as presented in Fig. 2. The treadmill group improved DT gait velocity by 4.2%, and the physiotherapy group by 8.3% respectively. Interaction effects were not observed.

### Secondary outcome—gait parameters—single task, dual task and dual task costs

Almost all gait parameters improved significantly in both groups over time during dual task performance: stride length (treadmill 4.6 / physiotherapy 5.5%), swing time (0.9/1.1%), stance time (0.5/0.6%), toe-off angle (1.7/2.5%), heel-strike angle (11.7/10.4%) and max. toe clearance (6.5/8.5%). However, interaction effects in favor of one intervention group were not detected (*p* = 0.172–0.994). In Table 2, all evaluated DT gait parameters are presented. In ST, gait velocity and stride length significantly increased in both groups as expected (Table 3).

**Table 2:** Gait parameters during dual task

Means	Descriptive								Repeated Measures ANOVA								
Dual task gait parameters	Physiotherapy *n* = 51				Treadmill *n* = 49				Interaction time × Group			Main effect time			Main effect time group		
	Base M	SD	Post M	SD	Base M	SD	Post M	SD	*F* (1;98)	*p*	*η*^2^	*F* (1;98)	*P*	*η*^2^	*F*(1;98)	*p*	*η*^2^
Stride Time in s	1.15	0.14	1.12	0.11	1.14	0.10	1.16	0.14	0.259	0.612	0.003	0.259	0.612	0.003	0.551	0.46	0.006
Swing Time in %	36.11	1.40	36.50	1.26	35.95	1.47	36.28	1.67	0.056	0.814	0.001	8391	**0.005**	0.079	0.506	0.479	0.005
Stance Time in %	63.89	1.40	63.50	1.26	64.05	1.47	63.72	1.67	0.056	0.814	0.001	8391	**0.005**	0.079	0.506	0.479	0.005
Stride Length in cm	136.19	17.51	143.72	16.69	133.11	13.76	139.23	16.01	0.344	0.559	0.003	32.551	**0.001**	0.249	1.609	0.208	0.016
Max Lateral Excursion in cm	5.06	1.40	5.18	1.21	4.89	1.33	4.80	1.33	1.117	0.293	0.011	0.017	0.896	0.000	1.235	0.269	0.012
Gait Velocity in m/s	1.20	0.20	1.30	0.20	1.18	0.16	1.23	0.20	1.891	0.172	0.019	19.754	**0.001**	0.168	2033	0.157	0.02
TO Angle in °	− 66.47	5.93	− 68.14	6.00	− 65.29	6.42	− 66.38	6.62	0.477	0.492	0.005	10.948	**0.001**	0.100	1.558	0.215	0.016
HS Angle in °	15.63	5.59	17.25	5.05	13.80	4.65	15.41	5.29	< 0.000	0.994	< 0.000	12.739	**0.001**	0.115	3895	0.051	0.038
Max. Toe Clearance in cm	8.78	2.55	9.53	2.41	8.82	2.55	9.39	2.52	0.219	0.641	0.002	12.496	**0.001**	0.113	0.010	0.920	0.000

Numbers in bold indicate significance

*TO*  toe-off, *HS*  heel strike, *M*  mean, *SD* standard deviation, *Base* baseline, *Post*  post-test

**Table 3:** Single task gait parameters

Means	Descriptive								Repeated measures ANOVA								
Single task gait parameters	Physiotherapy *n* = 51				Treadmill *n* = 49				Interaction time × group			Main effect time			Main effect time group		
	Base M	SD	Post M	SD	Base M	SD	Post M	SD	*F* (1;98)	*p*	*η*^2^	*F* (1;98)	*P*	*η*^2^	*F*(1;98)	*p*	*η*^2^
Stride time in s	1.07	0.07	1.05	0.06	1.08	0.06	1.08	0.07	3.078	0.082	0.030	1.84	0.18	0.02	1.50	0.23	0.015
Swing time in %	36.89	1.30	37.01	1.09	36.78	1.39	36.64	1.49	1825	0.18	0.018	0.028	0.87	0.001	0.932	0.34	0.009
Stance time in %	63.11	1.30	62.99	1.09	63.22	1.39	63.36	1.49	1825	0.18	0.018	0.028	0.869	0.001	0.932	0.337	0.009
Stride length in cm	146.55	14.73	150.43	15.02	143.99	12.18	146.47	13.54	0.53	0.468	0.005	10.767	**0.001**	0.099	1553	0.216	0.016
Max lateral excursion in cm	5.32	1.35	5.29	1.23	4.93	1.23	5.06	1.50	0.579	0.449	0.006	0.18	0.672	0.002	1629	0.205	0.016
Gait velocity in m/s	1.37	0.15	1.43	0.15	1.34	0.12	1.37	0.16	1.384	0.242	0.014	8493	**0.004**	0.08	3.25	0.074	0.003
TO angle in °	− 70.77	466	− 71.25	5.07	− 69.00	5.53	− 69.29	582	0.095	0.759	0.001	1.614	0.207	0.016	3406	0.068	0.034
HS angle in °	17.48	5.11	18.80	4.51	16.54	4.09	18.40	4.92	0.478	0.491	0.005	16.571	**0.001**	0.145	0.623	0.432	0.006
Max. toe clearance in cm	9.74	2.30	10.35	2.34	9.81	2.10	10.52	2.04	0.093	0.761	0.001	16.79	**0.001**	0.146	0.084	0.772	0.001

Numbers in bold indicate significance

*TO* toe-off, *HS* heel strike, *M* mean, *SD*  standard deviation, *Base* baseline, *Post* Posttest

For all parameters except stride time and stance time (in %), negative means indicate that patients show a worsening of gait parameters under DT condition compared to ST condition. We observed a significant improvement over time for DT costs in gait velocity (*p* < 0.01) for both intervention groups. Swing time in % (*p* < 0.001), stance time in % (*p* < 0.001), and TO angle (*p* < 0.002) improved significantly as well. No significant interactions were observed for time and group and no main effect of group on any of the DT costs parameters. All results for DT costs are presented in Table 4.

**Table 4:** Dual task costs in % for spatiotemporal gait parameters

Means	Descriptive								Repeated measures ANOVA								
Dual task costs gait parameters	Physiotherapy *n* = 51				Treadmill *n* = 49				Interaction time × Group			Main effect time			Main effect time group		
	Base M	SD	Post M	SD	Base M	SD	Post M	SD	*F* (1;98)	*p*	*η*^2^	*F* (1;98)	*P*	*η*^2^	*F*(1;98)	*p*	*η*^2^
Stride time in s	7.05	12.54	6.44	10.81	6.16	6.53	7.25	9.79	1.02	0.32	0.01	0.081	0.78	0.001	0.00	0.980	0.001
Swing Time in %	− 2.09	2.90	− 1.36	2.56	− 2.24	2.56	− 0.95	2.86	0.85	0.36	0.009	11.19	**0.001**	0.10	0.078	0.781	0.001
Stance time in %	1.25	1.72	0.81	1.54	1.33	1.52	0.57	1.59	0.86	0.36	0.009	11.81	**0.001**	0.11	0.10	0.750	0.001
Stride Length in cm	− 7.16	6.40	− 4.44	6.17	− 7.58	5.10	− 4.96	6.24	6.24	0.939	0.001	16.646	**0.001**	0.145	0.218	0.642	0.002
Max lateral excursion in cm	− 4.11	17.46	− 0.79	14.80	0.03	16.28	− 3.12	18.80	2035	0.157	0.02	0.001	0.97	0.001	0.131	0.720	0.001
Gait velocity in m/s	− 12.28	10.72	− 9.36	10.40	− 12.47	8.66	− 10.43	11.21	0.19	0.66	0.002	6124	**0.015**	0.06	0.122	0.73	0.001
TO angle in °	− 6.14	4.72	− 4.42	4.29	− 5.45	3.90	− 4.25	4.28	0.33	0.57	0.003	10.476	**0.002**	0.1	0.34	0.56	0.003
HS ANGLE IN °	− 6.19	51.57	− 5.70	31.26	− 17.99	13.94	− 17.52	18.32	0.00	0.99	0.001	0.035	0.85	0.001	3.89	0.051	0.038
Max. toe clearance in cm	− 10.2	14.88	− 8.09	12.52	− 10.80	12.70	− 11.47	16.09	0.82	0.37	0.008	0.220	0.64	0.002	0.703	0.404	0.007

Numbers in bold indicate significance

*TO* toe-off, *HS*  heel strike, *M* mean, *SD* standard deviation, *Base* baseline, *Post* post-test

### Secondary outcomes—UPDRS III & BBS & 2 minute walk test

UPDRS-III scores significantly decreased from baseline to post-test in both groups. Interaction of time and group did not reach significance level (*F*(1,98) = 3.82; *p* < 0.053; partial *η*^2^ = 0.38), but the main effect of time changed significantly (*F*(1,98) = 371.64; *p* < 0.0001; partial *η*^2^ = 0.791). A main effect of group was not observed.

BBS scores showed an incline over time for both interventions. Interaction effects did not reach significance level (P > 0.05), but showed improvements over time (*F*(1,98) = 38.927; *p* < 0.0001; partial *η*^2^ = 0.284). A significant effect for group was not present.

**Fig. 3:**
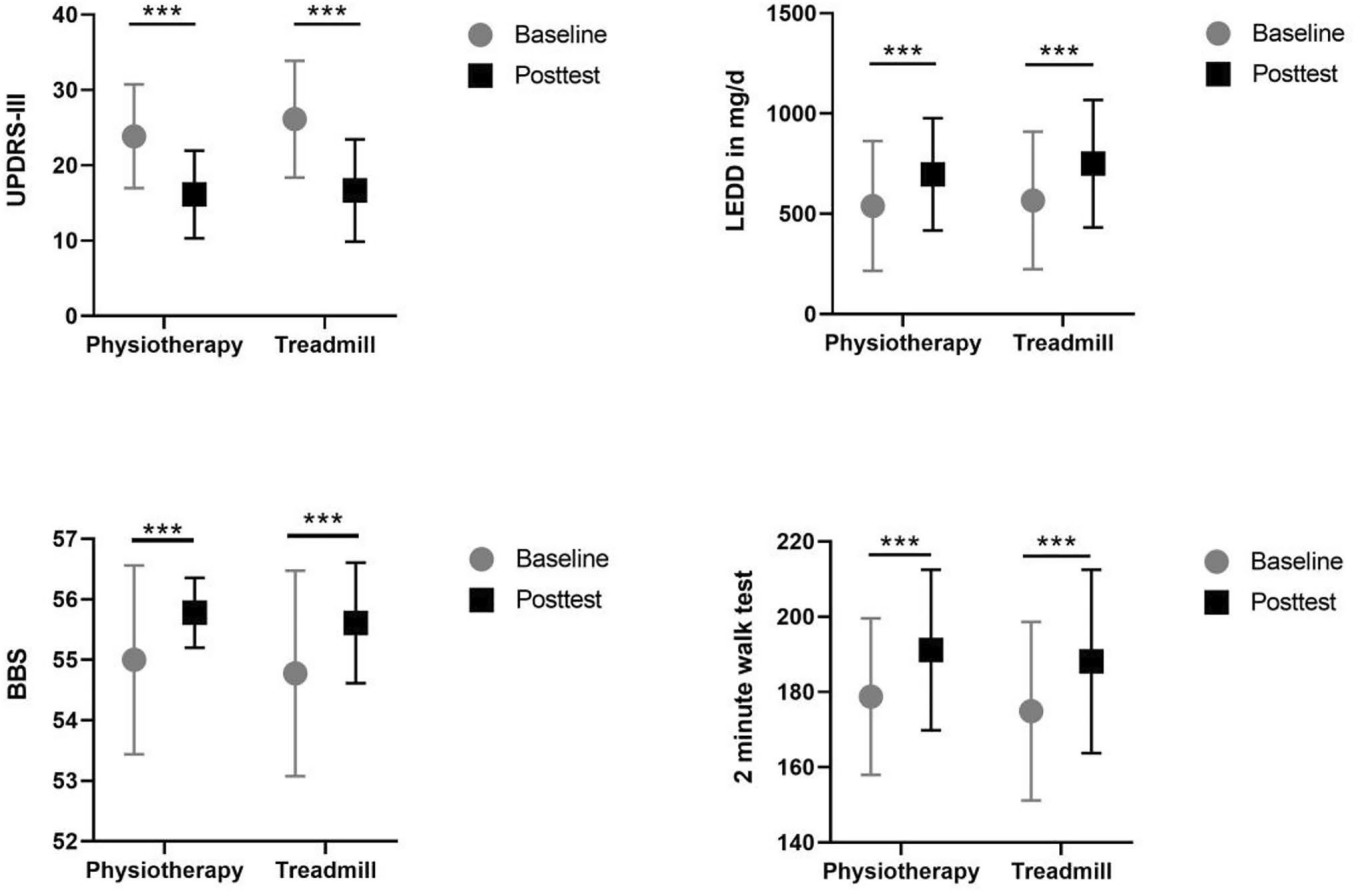
Secondary outcome measures: change of UPDRS-III (top left), Berg Balance Scale (BBS, bottom left) and walking distance in 2 min (bottom right). In addition, Levodopa Equivalent Daily Dose (LEDD, top right) is presented

The walking capacity (2-min walk test) showed significant improvements in walking distance over 2 min (*F*(1,98) = 59.932; *p* < 0.0001; *η*^2^ = 0.379) in both intervention groups. There was no main effect of group or group and time interaction detected. All time effects described above are visualized in Fig. 3.

## Discussion

The aim of this study was to investigate the impact of individualized physiotherapy and treadmill training on gait during dual task performance in PD patients with mild to moderate motor deficits. The main finding of this study was that both interventions significantly improved gait velocity and the majority of gait parameters during dual task walking as well as UPDRS-III scores and walking capacity. Importantly, standardized treadmill training did not yield a significantly larger effect than individualized physiotherapy.

### Primary outcome—gait velocity

Both intervention groups showed a significant increase in gait velocity during dual task. Gait velocity served as primary outcome measure for most studies concerning dual task walking and dual task training (Brauer and Morris 2010; De Freitas Tb Ms et al. 2020; Galletly and Brauer 2005; Gassner et al. 2017; O'Shea et al. 2002; Raffegeau et al. 2019; Rochester et al. 2008; Strouwen et al. 2019; Strouwen et al. 2017; Wollesen et al. 2021; Yogev et al. 2005; Yogev-Seligmann et al. 2012). It is one of the major parameters classifying the influence of dual task on gait and therefore an important factor in rating improvement due to intervention. Both interventions resulted in clinically relevant gait speed improvements that were minimal (5–6 cm/s) to moderate (around 14 cm/s) (Hass et al. 2014; Yang et al. 2019). With 10 cm/s, gait speed in the physio group improved more than in the treadmill group (5 cm/s). This could be due to the fact that a few days (at least the first 3 but up to 5 days) are needed for patients to reach their overground speed on the treadmill before they start improving (Godi et al. 2020). As treadmill intervention in the present study was limited to 14 days, this intervention was probably not given enough time to develop its full potential. When comparing our results to studies that explicitly trained dual task walking (walking and training a cognitive task simultaneously), our results align with the improvement of 8.3 cm/s in previous studies (Strouwen et al. 2017). There are two studies observing a higher level of improvement by 25 cm/s (Yogev-Seligmann et al. 2012) and 14.7 cm/s (Yang et al. 2019); however, both were conducted with a very small sample size (*n* = 7, *n* = 6 per group) and must therefore be interpreted cautiously. Notably, even though the improvements were comparable, the baseline gait speed of the present study was significantly higher compared to other studies mentioned above indicating that our PD patients were less impaired.

These data suggest that training of a second cognitive task may not be required to yield benefits on dual task walking. However, in many studies investigating the effect of dual task training on gait that train a second cognitive task, benefits were preserved over time after the end of the intervention [2,26,38]. In the present study, follow-up visits were not conducted due to the clinical setting in which patients are discharged from the hospital after the intervention period. It is unclear yet if training of a secondary cognitive task besides providing physiotherapy or treadmill walking might yield an even higher effect. Further research is necessary to investigate this question in more depth.

### Secondary outcomes—other gait parameters and dual task prioritization

This study detected significant improvements in various gait parameters under dual task conditions. The most prominent being stride length as another gait parameter that is usually measured, worsens during dual tasking (Bond and Morris 2000; Galletly and Brauer 2005; Morris et al. 1996; O'Shea et al. 2002) and shows improvement after dual task training interventions similar to our results (Geroin et al. 2018; Yang et al. 2019). The large majority of dual task gait parameters were positively affected by the interventions and herewith objectively confirm the improvements detected by the rater-dependent clinical score UPDRS-III. This is in line with previous studies that have shown positive effects on these gait parameters during single task caused by physiotherapy as well as treadmill walking. (Gassner et al. 2019; Radder et al. 2020). A more fine-granular effect of one intervention by objective, sensor-based measures was not detected.

Task prioritization during dual task often is an issue when drawing conclusions from studies as the observed improvement may reflect rather a shift in priority. When facing a dual task, PD patients are considered to use a “posture second” strategy, prioritizing the cognitive task or no specific task at all (Bloem et al. 2006). As we did not instruct patients to prioritize one task, it is possible that patients focused on the cognitive task at baseline and therefore showing severe losses in gait performance while prioritizing gait during follow-up, resulting in better gait but lower cognitive performance. However, as the number of completed and correct calculations during serial 3 subtractions at baseline and post-test remained equal and all described changes in gait above are clinically relevant, we conclude that the observed changes may be attributed to an actual, clinically measurable improvement. There is evidence in young people showing that treadmill walking facilitated cognitive performance in contrast to overground walking (Penati et al. 2020). This encourages the aim to further investigate treadmill walking as an external stimulus that possibly supports dual-task walking.

### Secondary outcomes—clinical outcomes

Results of this study show significant improvements in UPDRS-III and walking capacity similar to other studies testing the effect of physiotherapy and treadmill training interventions (Gassner et al. 2019; Paz et al. 2019; Pellecchia et al. 2004; Radder et al. 2020). When compared to the LSVT BIG training (derived from the Lee Silverman Voice Treatment) with longer intervention periods (16 sessions for 1 h each over 4 weeks) that has been proven to be effective and decreases the UPDRS- III score by 5 points (Ebersbach et al. 2010) or 7 points (Ebersbach et al. 2015), our interventions reached a similar or even higher effect. Furthermore, with mean improvements by 9 points (treadmill) and 8 points (physio), both intervention groups are above the commonly used cutoff score of 5 points that indicates the minimal clinically important change (Schrag et al. 2006).

In this context, it needs to be considered that adjustments of medication occurred in both intervention groups (see limitations); therefore, improvements in the UPDRS motor score resulted from a combined therapy approach.

Patients balance (BBS) improved significantly in both groups. This finding is similar to several studies reporting improved BBS for both, physiotherapy (Yitayeh and Teshome 2016) as well as treadmill training (Ganesan et al. 2014). As balance impairment is a major factor in dual task walking deficits (Brauer et al. 2011; Woollacott and Shumway-Cook 2002), balance is a common outcome measure for dual task interventions. Observed improvements in dual task studies using BBS are consistent with our results (De Freitas Tb Ms et al. 2020; Silva and Israel 2019). Furthermore, walking capacity measured by the 2-min walking test improved significantly. Compared to studies assessing the walking capacity in a 6-min walking test in physiotherapy and treadmill walking, our results show similar improvements (Paz et al. 2019; Radder et al. 2020).

### Adverse events and therapeutic options

Practicability and acceptance of both interventions were generally good. Compliance was very high throughout the study with 100 out of 105 participants completing the intervention (n(physio) = 51, n(treadmill) = 49). Only one not to the intervention-related adverse event was recorded, all other dropouts were due to external causes (see Fig. 1), suggesting the general safety of our interventions. As mentioned above, dual-tasking in PD patients is often associated with an increased risk of falling. Therefore, having safer training options, such as physiotherapy and treadmill training, is very valuable especially for PD patients with a history of falling or fear of falling. Since both interventions have shown in the present study to be similarly effective, patients may be treated with one of these therapeutic interventions based on, e.g., personal preferences or local treatment settings to provide one or the other intervention. Furthermore, effective treatment is not limited to one specific intervention and therefore there would be more therapeutic opportunities for patients to have a timely access for treatment. Nevertheless, dual-tasking is essential for the daily life of PD patients and should therefore be considered in future interventional studies.

## Limitations

Adjustment of medication occurred during interventions as it is part of the standardized ‘Parkinson’s Disease Multidisciplinary Rehabilitation’ program in Germany, which might have influenced motor symptoms and therefore measurements of the post-test. Both groups increased LEDD (Physio + 158 mg/d, Treadmill + 183 mg/d) which has been shown to improve UPDRS-III by about 4 points (Hauser et al. 2009). Furthermore, both intervention groups received additional group therapy that might have yielded benefits. Thus, improvements in UPDRS-III in this study by 8–9 points may reflect the effect of the combined therapy approach (medication + physical activity (group therapy + intervention)). An isolated observation of the effects of physiotherapy and treadmill walking was not possible under the given inpatient setting. To further investigate this effect, future studies should maintain stable medication during intervention. In addition, future studies should include a follow-up visit to investigate whether benefits sustain over a longer period of time.

## Conclusion

In conclusion, this study presented that individualized physiotherapy and treadmill training over 14 days significantly improved gait speed and additional gait parameters during dual task walking as well as clinical parameters and walking capacity in patients with mild to moderate PD. However, structured treadmill walking did not show significantly more improvements than individualized physiotherapy. Our data suggest that both interventions are able to improve dual task walking and therefore support safe and independent walking in everyday life of PD patients. This may lead to new and more tailored therapeutic options. To further verify this approach, we suggest follow-up studies to investigate whether the benefits of the conducted interventions are sustainable.
